# Donor-Derived Myeloid Sarcoma in Two Kidney Transplant Recipients from a Single Donor

**DOI:** 10.1155/2015/821346

**Published:** 2015-04-21

**Authors:** Amudha Palanisamy, Paul Persad, Patrick P. Koty, Laurie L. Douglas, Robert J. Stratta, Jeffrey Rogers, Amber M. Reeves-Daniel, Giuseppe Orlando, Alan C. Farney, Michael W. Beaty, Mark J. Pettenati, Samy S. Iskandar, David D. Grier, Scott A. Kaczmorski, William H. Doares, Michael D. Gautreaux, Barry I. Freedman, Bayard L. Powell

**Affiliations:** ^1^Department of Internal Medicine, Section on Nephrology, Wake Forest School of Medicine, Winston-Salem, NC 27103, USA; ^2^Department of Pathology, Wake Forest School of Medicine, Winston-Salem, NC 27103, USA; ^3^Department of Internal Medicine, Section on Hematology and Oncology, Comprehensive Cancer Center of Wake Forest University, Winston-Salem, NC 27103, USA; ^4^Department of General Surgery, Wake Forest School of Medicine, Winston-Salem, NC 27103, USA; ^5^Department of Pharmacy, Wake Forest School of Medicine, Winston-Salem, NC 27103, USA

## Abstract

We report the rare occurrence of donor-derived myeloid sarcoma in two kidney transplant patients who received organs from a single deceased donor. There was no evidence of preexisting hematologic malignancy in the donor at the time of organ recovery. Both recipients developed leukemic involvement that appeared to be limited to the transplanted organ. Fluorescence *in situ* hybridization (FISH) and molecular genotyping analyses confirmed that the malignant cells were of donor origin in each patient. Allograft nephrectomy and immediate withdrawal of immunosuppression were performed in both cases; systemic chemotherapy was subsequently administered to one patient. Both recipients were in remission at least one year following the diagnosis of donor-derived myeloid sarcoma. These cases suggest that restoration of the immune system after withdrawal of immunosuppressive therapy and allograft nephrectomy may be sufficient to control HLA-mismatched donor-derived myeloid sarcoma without systemic involvement.

## 1. Introduction

Donor-derived malignancies occur in less than 0.1% of kidney transplant recipients [1]. However, increased utilization of older donors in the last decade has the potential to increase recipient risk for the development of donor-derived malignancy [2]. Although the most common donor-derived cancers after kidney transplantation are renal cell carcinomas, various types of donor-derived malignancies have been reported [3]. We report the rare occurrence of two cases of myeloid sarcoma that developed following kidney transplantation from a single deceased donor, in which the donor origin of the neoplastic clone was verified by molecular genotyping in each case.

## 2. Case Report: Donor

A 38-year-old female nursing home resident with a body mass index (BMI) of 44 kg/m^2^ and bilateral sequential lower extremity amputations was found unresponsive and pulseless for an unknown period of time and was immediately intubated at the scene. Cardiopulmonary resuscitation was initiated with restoration of circulation. Following transfer to a nearby acute care hospital, neurological examination revealed apnea and a computerized tomographic (CT) scan of the head revealed diffuse cerebral edema. She was pronounced dead by brain death criteria and the family consented to organ donation.

Past medical history provided by the family and skilled nursing facility revealed that the patient had been hypertensive for 3 years and had a history of diabetes mellitus for 20 years. She also smoked tobacco and had resultant chronic obstructive pulmonary disease. However, she did not have any history of cancer or chronic weight loss. On the day of admission, the white blood cell count was 4800 cells/microL (differential 83% neutrophils, 4% monocytes, and 13% lymphocytes), hemoglobin was 10.3 grams/dL, and platelet count was 125,000/microL. Peripheral blasts were not detected on a blood smear. Both kidneys were recovered for transplantation. No other organs were used (the liver was not recovered because of steatosis). Although an autopsy was not performed, a preimplantation kidney biopsy revealed changes that were consistent with long-standing diabetes mellitus.

## 3. Recipient 1

The first kidney transplant recipient was a 72-year-old European American male with a history of type 2 diabetes mellitus and hypertension culminating in end-stage renal disease (ESRD) who had been on hemodialysis for 2 years. He had previously undergone thyroidectomy for papillary adenocarcinoma of the thyroid and radical prostatectomy with pelvic lymphadenectomy for prostate cancer. He also had a remote history of tobacco use. The recipient and donor were a two-human leukocyte antigen (HLA) mismatch. During the transplant procedure, he received intravenous induction therapy with a single intraoperative dose of alemtuzumab (30 mg) and dexamethasone (100 mg). Cold ischemia time was 13.5 hours; the kidney reperfused well and appeared anatomically normal. A reperfusion biopsy of the renal cortex showed mild to moderate donor transmitted chronic changes and acute tubular injury. Following transplantation, maintenance immunosuppression was initiated and included tacrolimus and mycophenolic acid. The patient experienced slow graft function but did not receive any hemodialysis treatments postoperatively. A one-month surveillance biopsy of the renal allograft revealed residual acute tubular injury and donor transmitted diffuse and nodular diabetic glomerulosclerosis. Serum creatinine levels subsequently stabilized in the 2.5–3.0 mg/dL range.

**Figure 1:**
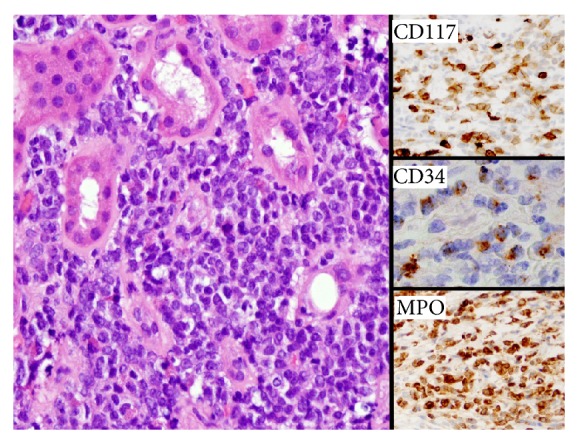
Recipient 1, kidney biopsy: A diffuse proliferation of immature myeloid cells is seen dissecting between renal tubules. Immunohistochemical analysis for blasts (CD117 and CD34) and myeloid lineage (MPO, myeloperoxidase) were strongly positive.

Two months following transplantation, he was readmitted for dyspnea attributed to bilateral pneumonia and pulmonary edema thought to be cardiac in nature. He improved with diuresis and antibiotic therapy. Recurrent dyspnea and pulmonary edema developed four months following transplantation at which time he was readmitted and found to have acute kidney injury (serum creatinine level 4.5 mg/dL) and volume overload. An ultrasound examination of the renal allograft demonstrated a significant increase in volume of the transplanted kidney and elevated resistance indices. A subsequent renal allograft biopsy revealed diffuse parenchymal infiltration with immature mononuclear cells. Immunohistochemical studies revealed CD34, CD117, and myeloperoxidase (MPO) positive blasts consistent with a diagnosis of myeloid sarcoma (Figure 1). Fluorescence* in situ* hybridization (FISH) studies showed normal chromosomes and confirmed that 93% of the cells in the biopsy were of donor (female, XX) origin, suggesting a donor-derived myeloid sarcoma transmitted with the transplanted kidney. At the time of kidney biopsy, the white blood cell count was 2800 cells/microL, hemoglobin was 11 grams/dL, and platelet count was 95,000/microL. There were no peripheral blasts detected and a bone marrow biopsy was negative for leukemic involvement. Positron Emission Tomography/CT (PET/CT) did not reveal any other foci of involvement beyond the renal allograft. Conventional metaphase cytogenetic analysis of the bone marrow biopsy revealed a normal male karyotype with no apparent leukemic involvement.

Initial treatment included planned embolization of the transplant renal artery to induce allograft infarction followed by uneventful nephrectomy performed on the following day. Immunosuppression was immediately discontinued. Direct tissue sample following nephrectomy showed 5.5% XY and 94.5% XX chromosomes. He subsequently completed induction therapy with one cycle of cytarabine and daunorubicin (7 + 3). A repeat bone marrow biopsy performed 5 months following the initial diagnosis did not reveal any evidence of disease. He remained in remission and on dialysis for another 8 months before sustaining a cardiovascular death 13 months following nephrectomy.

## 4. Recipient 2

The second kidney transplant recipient was a 77-year-old European American woman with ESRD secondary to interstitial nephritis who had been on hemodialysis for 2 years. She had a history of a prior failed renal transplant at another center secondary to early renal artery thrombosis that resulted in allograft nephrectomy. The recipient and donor were a three-HLA mismatch. Cold ischemia time was 28 hours; the kidney reperfused well and appeared normal anatomically. A reperfusion biopsy of the renal cortex showed mild to moderate donor transmitted chronic changes and acute tubular injury. Induction therapy consisted of alemtuzumab and dexamethasone with tacrolimus and mycophenolic acid maintenance immunosuppression. She experienced immediate graft function and serum creatinine levels eventually stabilized in the 1.4–1.7 mg/dL range. She was admitted with fever two weeks following kidney transplantation and was successfully treated for a urinary tract infection. Three weeks following transplantation, she underwent a surveillance allograft biopsy that revealed recovering acute tubular injury, donor transmitted nodular diabetic glomerulosclerosis, and hyalinosis.

The patient was readmitted 4 months following transplantation to another facility for acute kidney injury (serum creatinine level > 4.0 mg/dL) and recurrent urinary tract infections. A kidney biopsy showed acute and chronic thrombotic microangiopathy and immunosuppression was changed from tacrolimus to a cyclosporine-based regimen. However, upon further review, atypical cells were noted in the biopsy and renal function did not improve with conversion to cyclosporine. By this time, the diagnosis of myeloid sarcoma had been confirmed in Recipient 1 (above), who received the mate kidney from the same donor. Recipient 2 was notified of these findings and she opted for allograft nephrectomy. Laboratory analysis at the time of readmission to our facility revealed allograft dysfunction with a serum creatinine level of 4.3 mg/dL. Her white blood cell count was 2700 cells/microL, hemoglobin was 8.3 grams/dL, and platelet count was 109,000/microL. There were no peripheral blasts and PET/CT scan did not reveal any fluorodeoxyglucose (FDG) avid lesions other than the renal allograft. The patient refused a bone marrow biopsy.

**Figure 2:**
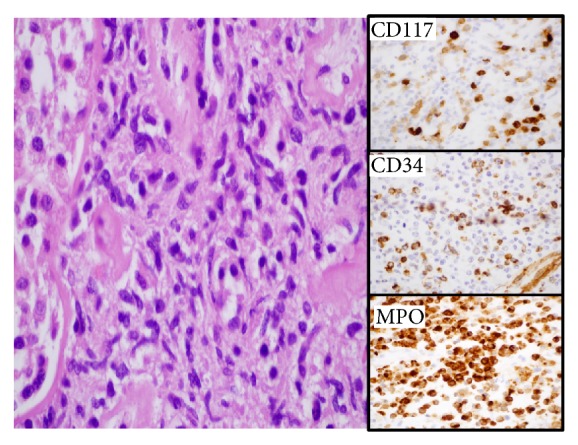
Recipient 2, kidney biopsy: A diffuse proliferation of immature myeloid cells is seen dissecting between renal tubules. Immunohistochemical analysis for blasts (CD117 and CD34) and myeloid lineage (MPO, myeloperoxidase) were strongly positive.

**Figure 3:**
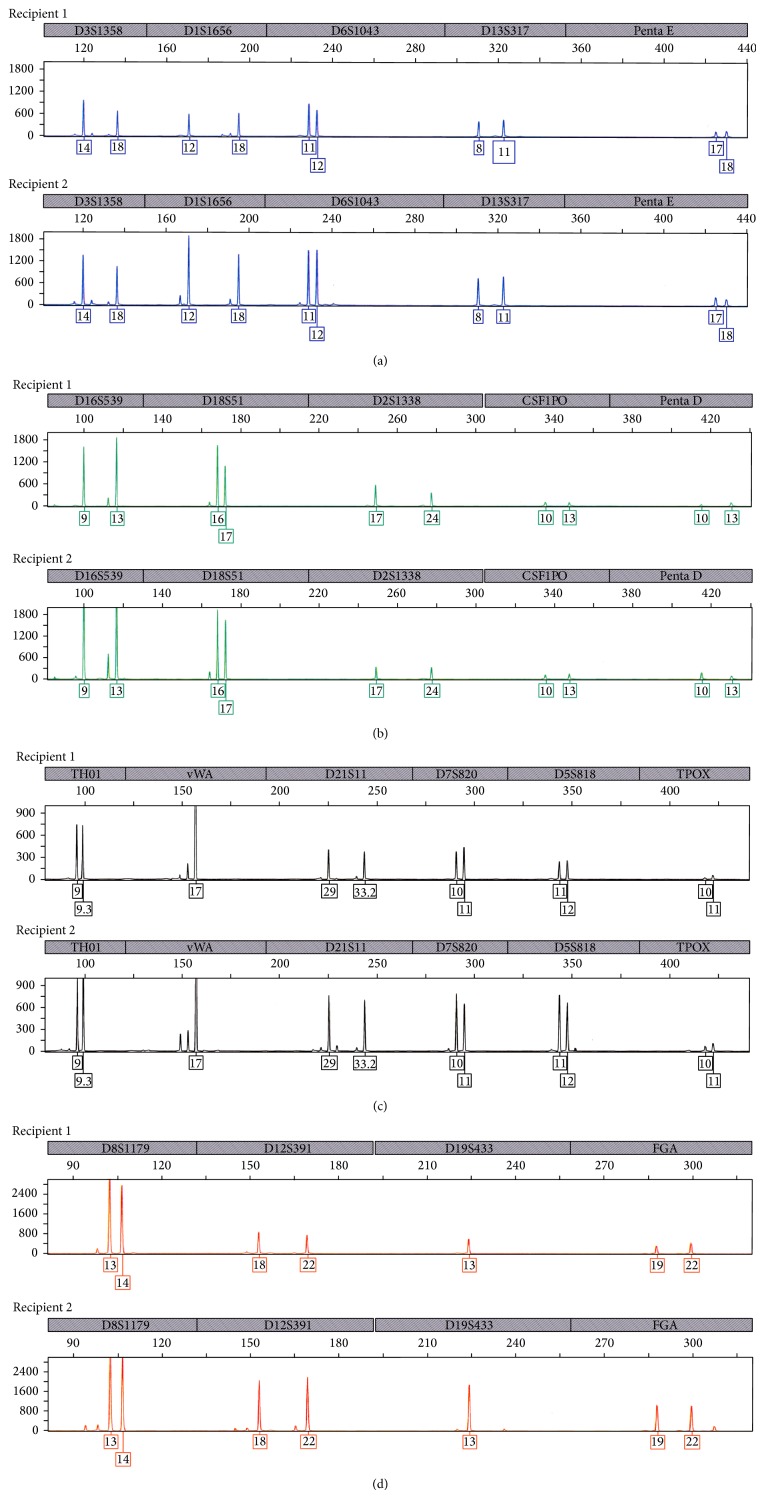
Molecular genotyping of Recipients 1 and 2 tumors for 21 informative markers (PowerPlex 21, Promega Corp).

Allograft nephrectomy was performed following planned preoperative embolization. Pathologic examination of the explant demonstrated a monotonous population of myeloid blasts morphologically identical to the first transplant recipient (Figure 2). Molecular genotyping analysis performed on renal tissue from each recipient yielded identical haplotypes and confirmed that both myeloid sarcomas were of donor origin (Figure 3). Immunosuppression was immediately withdrawn. The patient chose not to receive systemic chemotherapy and she remained in remission and on dialysis for another 18 months following nephrectomy before sustaining a cardiovascular death.

## 5. Discussion

This report describes the rare development of donor-derived myeloid sarcoma in two kidney transplant recipients from a single donor. Donor-derived leukemia has been reported after solid organ transplantation in liver transplant recipients and in a renal transplant recipient [4–6]. In these cases, the authors hypothesized that the most likely mechanism of disease was transformation of normal hematopoietic stem cells residing in the allograft into a malignant clone following transplantation. The authors favored this hypothesis given the absence of clinically evident disease in the donor prior to donation, a greater than two-year interval between transplantation and leukemic presentation, and the lack of involvement in recipients who received other organs from the same donor [4–6]. In the present report, although there was no evidence of preexisting hematologic malignancy in the donor, it appears most likely that a leukemic clone was transplanted through kidney tissue as both kidney transplant recipients were affected, disease was renal-limited, and both FISH and molecular genotyping analyses confirmed that the malignant cells were of donor origin.

It is remarkable that, despite partial HLA mismatch, the leukemic clone proliferated in both recipients. It has been reported that leukemic cells with normal karyotypes can display genomic instability in the form of uniparental disomy in myeloid cancers [7–9]. Vago et al. demonstrated that leukemic cells are able to elude antitumor donor T cells after haploidentical hematopoietic stem cell transplantation with infusion of donor T cells, by failing to express the mismatched HLA haplotype [10]. This effect leads to relapse of leukemia. It is plausible that a similar phenomenon occurred in these two recipients, whereby genomic instability of the transplanted leukemic clones resulted in decreased expression of mismatched HLA allowing leukemic cells to escape from alloreactive recipient T cells and proliferate. We also hypothesize that immunosenescence associated with the advanced age of the recipients and need for immunosuppressive drugs following transplantation impaired the recipient's immune response to a partially HLA-mismatched leukemic clonal proliferation.

As opposed to previous reports of leukemia after solid organ transplantation, this report demonstrates leukemic involvement of only the transplanted organs [4–6]. We believe that these cases may be the first reported cases of donor transmitted renal-limited myeloid sarcoma to two recipients from the same deceased organ donor. Both recipients lacked peripheral blasts and had negative PET/CT scans looking for systemic involvement, so a high index of suspicion is required to make this diagnosis. Bone marrow biopsy was also negative in the first recipient. The treatment regimens differed in each recipient based upon patient preference. Allograft nephrectomy and immediate withdrawal of immunosuppression were performed in both patients, while systemic chemotherapy was only administered to one. Both recipients remained in remission for at least one year following transplantation. These unusual cases suggest that restoration of the immune system after withdrawal of immunosuppressive therapy and allograft nephrectomy may be sufficient to control HLA-mismatched donor-derived myeloid sarcoma without systemic involvement.
